# Incidental diagnosis of tracheal trifurcation detected during general anesthesia from Nepal: a case report

**DOI:** 10.1097/MS9.0000000000002876

**Published:** 2025-01-31

**Authors:** Sandesh Gaire, Bibhush Shrestha, Ashish Acharya, Kritika Bhattarai, Santosh Basyal, Roshani Bhattarai

**Affiliations:** a Department of Cardiothoracic and Vascular Anesthesiology, Manmohan Cardiothoracic Vascular and Transplant Centre, Kathmandu, Nepal; b Nepal Medical College and Teaching Hospital, Kathmandu University, Kathmandu 44600, Nepal; c Shree Medical and Technical College, Chitwan, Nepal

**Keywords:** bronchoscopy, case report, general anesthesia, tracheal trifurcation

## Abstract

**Introduction and importance:**

Tracheal trifurcation is an uncommon condition characterized by a three-branched bronchial structure that can be directed either to the right or the left, most commonly on the right. It is associated with tracheobronchial anomalies, cardiovascular defects, and esophageal malformations. The intraoperative diagnosis of tracheal trifurcation is important when lung isolation is needed.

**Presentation of case:**

A three-branched structure at the carina was incidentally found in a 55-year-old female via a bronchoscope after the induction of general anesthesia when the lungs could not get isolated for a tricuspid valve replacement procedure, which was planned via right-sided thoracotomy. Initially, the patient complained of shortness of breath on exertion with concomitant multiple episodes of sore throat and fever. The patient was undergoing tricuspid valve replacement for rheumatic heart disease. There was no postoperative complication encountered.

**Discussion:**

Tracheal trifurcation or tracheal bronchus is an aberrant bronchus arising most commonly from the right side in the right upper bronchus between the carina and the cricoid cartilage. It is a very uncommon condition with most cases being asymptomatic. The condition has anesthetic implications, especially with the placement of endotracheal tube. These include lung field hypoxemia, shunting, and atelectasis.

**Conclusion:**

Through our case report, we aim to highlight the importance of viewing the bronchial morphology if the lung cannot be isolated after double-lumen endotracheal tube insertion. Hence, a careful assessment of both anatomical and physiological parameters of the bronchial tree and the lungs is to be done for such cases.

**HIGHLIGHTS**

- Tracheal trifurcation or tracheal bronchus is an aberrant bronchus arising most commonly from the right side in the right upper bronchus between the carina and the cricoid cartilage.
- It is a very uncommon condition. The incidence of the disorder is reported to be 1.08% with most cases being asymptomatic.
- Tracheal trifurcation has several anesthetic implications, especially with the placement of the endotracheal tube. The overall effects of endotracheal tube placement in tracheal bronchus can lead to hypoxemia, shunting, and atelectasis.
- Difficulty with double-lumen tube and lung isolation has been reported in cases of tracheal trifurcation. Our case also had difficulty in the isolation of the lungs. The reason behind this is aberrant right upper lobe takeoff, which makes this case rare and the first to be reported from Nepal.
- Our case report aims to highlight the importance of viewing the bronchial morphology if the lung cannot be isolated after double-lumen endotracheal tube insertion and the importance of a careful assessment of both anatomical and physiological parameters of the bronchial tree and the lungs.

## Introduction

There is no absolute definition of the disease; some authors define tracheal trifurcation as three bronchi that can be directed to either the right or left lung, while others only use the term when two of the three bronchi are directed toward the right lung^[1]^. The incidence of tracheal trifurcation is 1.08%^[2]^. Tracheal trifurcation is often associated with disorders like tracheobronchial anomalies, cardiovascular defects like atrioventricular septal defect, Tetralogy of Fallot (ToF), and esophageal malformations like esophageal atresia and tracheoesophageal fistula. These are responsible for clinical manifestations and earlier detection of the anomaly^[1]^. The preoperative diagnosis of tracheal trifurcation is important, especially when lung isolation is needed for certain procedures like tricuspid valve replacement. The left-sided double-lumen tube (DLT) can be used for most patients with a tracheal trifurcation^[2]^.

Here we intend to report a case of tracheal trifurcation, which was diagnosed in a female after inducing the general anesthesia. This is the first reported and diagnosed case of tracheal trifurcation detected during the general anesthesia form Nepal. This case report has been reported in accordance with the 2023 Surgical CAse REport (SCARE) Guidelines^[3]^.

## Case presentation

A 55-year-old married Hindu female from Kathmandu, Nepal, presented to our institution with a complaint of shortness of breath on exertion for 3 years. She had frequent episodes of sore throat with multiple episodes of fever in the past. The patient did not have any comorbidities like hypertension and diabetes mellitus. The patient had no relevant past and family history. There was no significant surgical or operative history mentioned by the patient.

The patient was present in an anxious state; however, the vitals were stable during the time of presentation. On examination of the cardiovascular system, a systolic murmur was heard on the left sternal border. The murmur was high-pitched and blowing in character.

**Figure 1.**
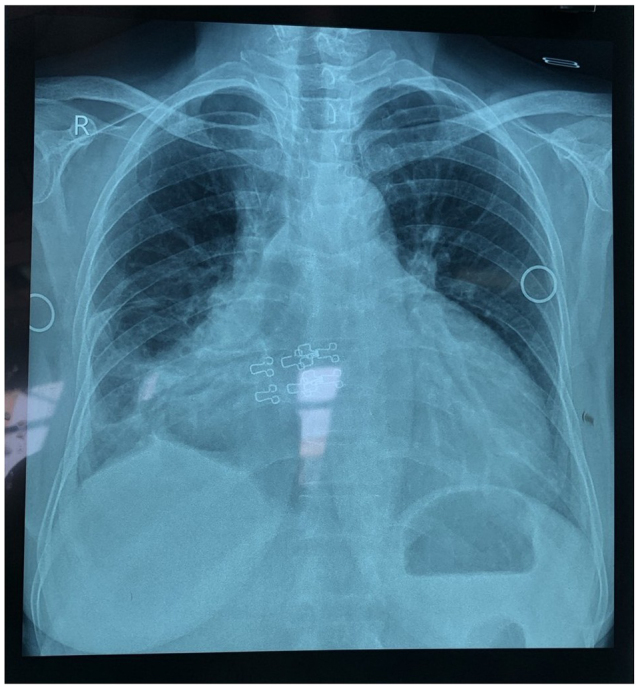
Showing a chest X-ray that depicts features of cardiomegaly.

A radiological investigation of a chest X-ray showed cardiomegaly as shown in Fig. 1.

**Figure 2.**
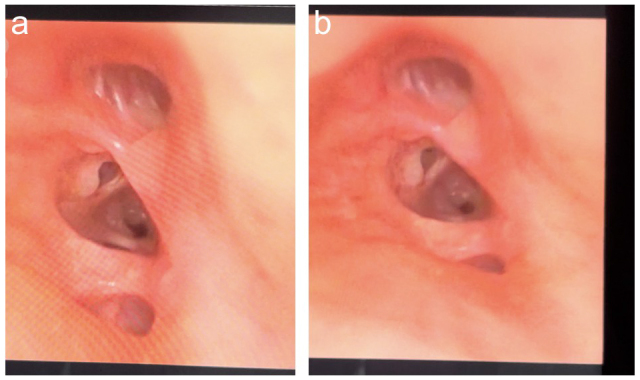
An image captured by a fiberoptic bronchoscope with a tracheal trifurcation at the right side of the carina.

Hence, a transthoracic echocardiography was performed, which revealed severe tricuspid regurgitation with a pressure gradient of 31 mm Hg and left ventricular ejection fraction of 60%. Mild pulmonary artery hypertension with a pulmonary artery systolic pressure of 31 + 10 = 41 mm Hg was noted.

The patient was then scheduled for tricuspid valve replacement via right-sided thoracotomy under general anesthesia.

At the routine preoperative evaluation, several blood tests were performed. The laboratory parameters showed: hemoglobin, 14.5 g/dl; a total count of 6000 cells/mm^3^; and platelets, 170 000 cells/mm^3^. The liver function test revealed: total bilirubin, 0.9 mg/dl; direct bilirubin, 0.2 mg/dl; indirect bilirubin, 0.4 mg/dl; alanine aminotransferase, 35 U/l; and aspartate aminotransferase, 40 U/l. Her serum urea and serum creatinine concentrations were 28 mg/dl and 0.8 mg/dl, respectively. The coagulation study showed prothrombin time / international normalized ratio (PT/INR): 13/1. Hence, her investigations did not show any abnormalities that would be expected to cause problems during the pre-, intra-, and postoperative periods. A preoperative computed tomography (CT) scan was advised but the patient did not comply due to her financial limitations.

Before anesthesia was induced adequate pre-oxygenation was done with 100% oxygen via a mask for 5 min. The patient underwent induction of general anesthesia with Midazolam 2 mg, Fentanyl 100mcg, Propofol 80 mg, and Rocuronium 50 mg. A left-sided 39-French double-lumen endotracheal tube (ET) was placed easily using direct laryngoscope. The oxygen saturation was maintained at 100%, an appropriate carbon dioxide waveform was noted on the capnograph after intubation, and the patient was noted to be hemodynamically stable.

The breath sounds were grossly equal on the bilateral lung field when tracheal cuff was inflated. When the tracheal cuff was inflated, both upper lung fields produced roughly similar breath sounds. After both cuffs were inflated, breath sounds on the right were significantly reduced, but not entirely gone, when the bronchial (left) lumen was clamped.

The left side’s breath sounds remained unaltered. Breath sounds on the left stopped when the tracheal (right) lumen was constricted, but they were still audible on the right. It was believed at this point that a right main-stem bronchial intubation had taken place instead of the intended left main-stem bronchial intubation. After being carefully taken out and put back in, the DLT produced the same auscultatory results.

Following the insertion of the fiberoptic bronchoscope down the tracheal lumen, the tracheal carina and a fairly modest right main-stem bronchus were visible. Further meticulous auscultation of all lung fields using clamping maneuvers showed that breath sounds stopped in the left upper and lower lung fields but remained over the right lung upon clamping of the tracheal lumen. Breath sounds were significantly reduced in the lower right lung fields, but only slightly reduced in the upper right lung field, and preserved in the left lung fields when the bronchial lumen was clamped.

When the fiberoptic bronchoscope was introduced into the Univent tube’s lumen, it was discovered that the carina had a trifurcation there rather than the typical bifurcation (Figure 2a and b).

The right upper lobe bronchus, which ended in a typical trifurcation that gave rise to the segmental bronchi, was the lumen that extended the furthest rightward according to the bronchoscope examination. However, the tracheal trifurcation could not be appreciated in the chest X-ray, as shown in Fig. 1.

Hence, a univalent tube with a bronchial blocker was used throughout the surgery due to difficulty in isolating the right side.

Throughout the surgery, anesthesia was maintained with isoflurane 1–1.5%, oxygen 2 L/min, air 2 L/min, and a continuous infusion of noradrenaline 0.08µg/kg/min. The duration of surgery was 260 min, and the patient’s respiratory status was stable. The patient was extubated after adequate spontaneous breathing. The patient was discharged home without complication following 4 days postoperatively and was satisfied with the management she received.

## Discussion

Tracheal bronchus is an aberrant or an accessory bronchus almost invariably arising from the right lateral wall of the trachea. It is an uncommon bronchial anomaly, and the study conducted by McLaughlin *et al*. reported tracheal bronchus as an incidental finding in 2% of children undergoing bronchoscopy.^[4]^ It can arise anywhere between the carina and the cricoid cartilage but is most frequently seen within 2 cm of the carina and has been reported as high as 6 cm above the level of carina^[5]^.

Tracheal trifurcation was unexpectedly found in the present case during visualization of carina with bronchoscope. The prevalence of tracheal trifurcation was found in 1 of 5000.^[6]^ A three-branched intra-tracheal structure or a tracheal bronchus commonly arises from the right upper lobe bronchus due to its embryological significance. The characteristic tracheal trifurcation belongs to class 3 of tracheal bronchus type^[7]^.

Several theories exist on the pathophysiology of tracheal bronchus with some including regression of the embryological tracheal buds or disruption of normal embryogenesis of trachea^[4]^. Tracheal bronchus has shown an association with tracheobronchial anomalies, esophageal atresia, and even atrioventricular septal defect. Literature has referenced the incidence of several cancers like squamous cell carcinoma, adenocarcinoma, and small cell carcinoma of lungs to be associated with patients who have an underlying smoking history and tracheal bronchus^[1,8]^. These associated anomalies were not present in our patient.

An anomaly like tracheal trifurcation can be diagnosed using a conventional CT scan and three-dimensional (3D) reconstruction prior to the induction of general anesthesia, especially in those patients undergoing surgical operations. However, a more advanced 3D reconstruction scan provides an easier visualization of the tracheobronchial anomaly than a conventional CT scan and is particularly helpful in demarcating the bronchial abnormalities. A recognition of this type of tracheal anomaly before intubation will help in the optimal positioning of the ET and further prevent postoperative complications like lobar collapse.^[9]^ However, in our case, the patient did not perform CT scan because of her financial restrictions, which we have regarded as one of the limitations of our case report.

The difficulty with DLT placement in a patient with an aberrant right upper lobe takeoff has been reported and referenced previously.^[10]^ Similarly, in our case, we too found it difficult to isolate the lungs. However, the reason behind this is aberrant right upper lobe takeoff, which makes this case rare and the first to be reported from Nepal.

The tracheal trifurcation cases have anesthetic implications too, with one including the placement of the ET. If the ET tube happens to be placed in one bronchus, then that lung field will only be ventilated. Similarly, if the ET tube is placed beyond the tracheal bronchus, all lung fields except the one supplied by the tracheal bronchus will be ventilated.^[7]^ Overall, endobronchial intubation in the tracheal bronchus can cause hypoxemia, shunting, and even atelectasis.^[5]^ The findings we got on auscultation can be explained by bronchoscope intervention. It has been strongly recommended to regularly use the bronchoscope to implant or confirm the right placement of a double-lumen ET^[10]^.

Most patients are asymptomatic. However, some cases presenting with cough, hemoptysis, or recurrent lung infections are not uncommon^[11]^.

Perioperative hypoxemia is a common but serious problem with several well-recognized etiologies. However, lobular collapse in association with anomalous bronchi in adult patients has been rarely reported^[12]^.

## Take away lessons

From our case, we strongly recommend reviewing the bronchial morphology through an anterior–posterior chest X-ray film prior to induction of general anesthesia, listening to the lung sounds bilaterally in all the lung fields, and doing a bronchoscopy to find out the position of the DLT. A preoperative conventional CT scan can also be considered beneficial to establish an early diagnosis. It is important to identify the anatomical variant of chest before any cardiac surgery in patients requiring intubation.

## Strength and limitation

This is the first reported rare case of tracheal trifurcation in a patient undergoing general anesthesia for a tricuspid valve replacement surgery. It was diagnosed at the time of general anesthesia, which itself is uncommon. However, further workup and management of such case could not be done in our institution. Similarly, a preoperative CT scan, which would have seen the bronchial anomaly, could not be performed prior to surgery due to the patient’s financial burden. These are the limitations of our case report. Nevertheless, we hope that the case report would add vital information to the existing literature on tracheal trifurcation.

## Conclusion

This case report highlights the incidental finding of tracheal trifurcation during general anesthesia in a female patient undergoing tricuspid valve replacement for severe tricuspid regurgitation due to rheumatic heart disease (RHD). Through our case report, we aim to give importance to a thorough respiratory or chest assessment and safety checks, especially after the induction of general anesthesia and endotracheal intubation. These involve a bilateral chest or lung field auscultation along with supportive radiological and bronchoscopy intervention. Such safety checks can minimize further respiratory complications.
